# Supplementary material for: Insights into the Role of the Microbiota and of Short-Chain Fatty Acids in Rubinstein–Taybi Syndrome
Source: Int J Mol Sci. 2021 Mar 31;22(7):3621. doi: 10.3390/ijms22073621 (PMC8037970; doi:10.3390/ijms22073621)
Supplement: Supplementary file 1 [file ijms-22-03621-s001.pdf]

International Journal of Molecular Sciences

Supplementary materials

Insights into the role of the microbiota and of short chain fatty acids in Rubinstein-Taybi syndrome

Elisabetta Di Fede[1,†], Emerenziana Ottaviano[1,†], Paolo Grazioli[1], Camilla Ceccarani[1,2], Antonio Galeone[3], Chiara Parodi[1], Elisa A. Colombo[1], Giulia Bassanini[1], Grazia Fazio[4], Marco Severgnini[2], Donatella Milani[5], Elvira Verduci [1,6], Thomas Vaccari[3], Valentina Massa[1,7,‡], Elisa Borghi[1,‡] and Cristina Gervasini[1,7,‡,*]

***Corresponding author:** cristina.gervasini@unimi.it; Tel.: +39-0250-3230-28

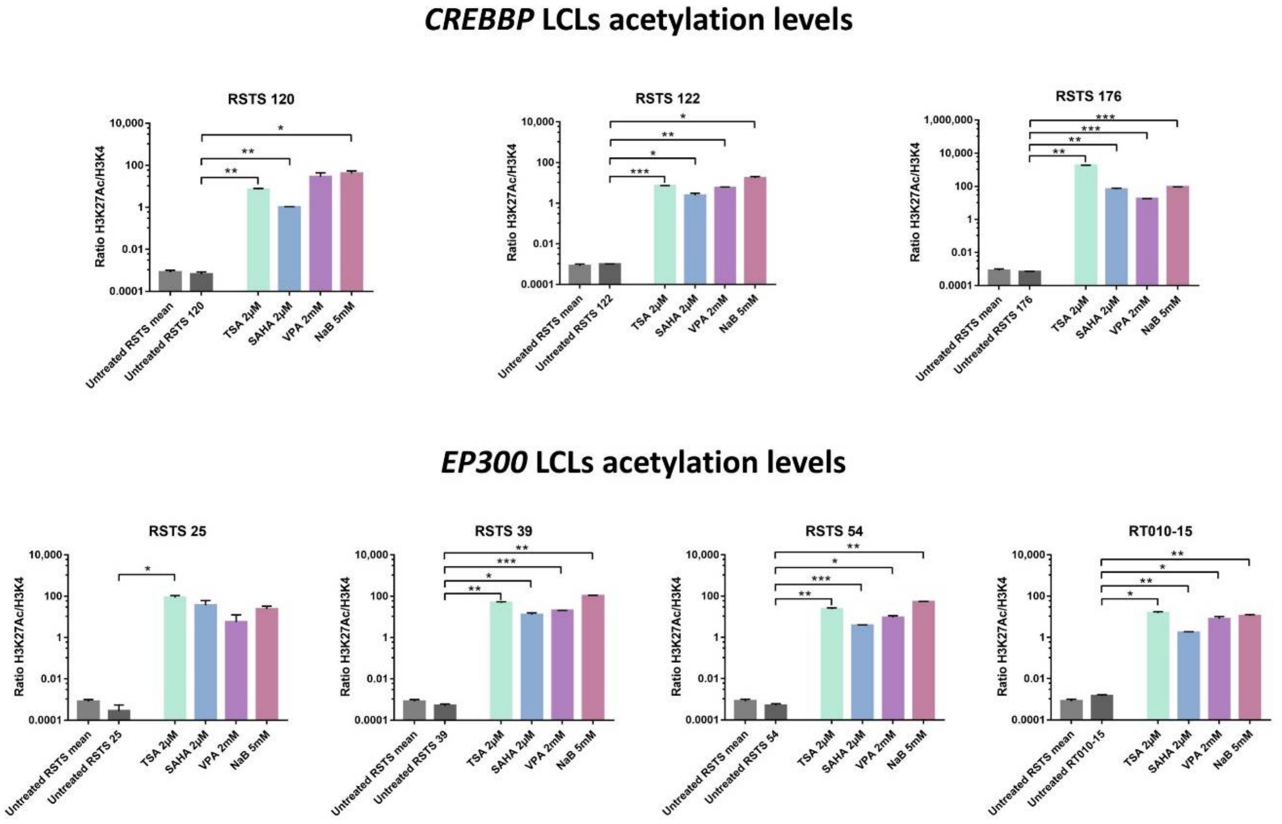

Figure S1. Insight on single-RSTS LCLs histone acetylation.

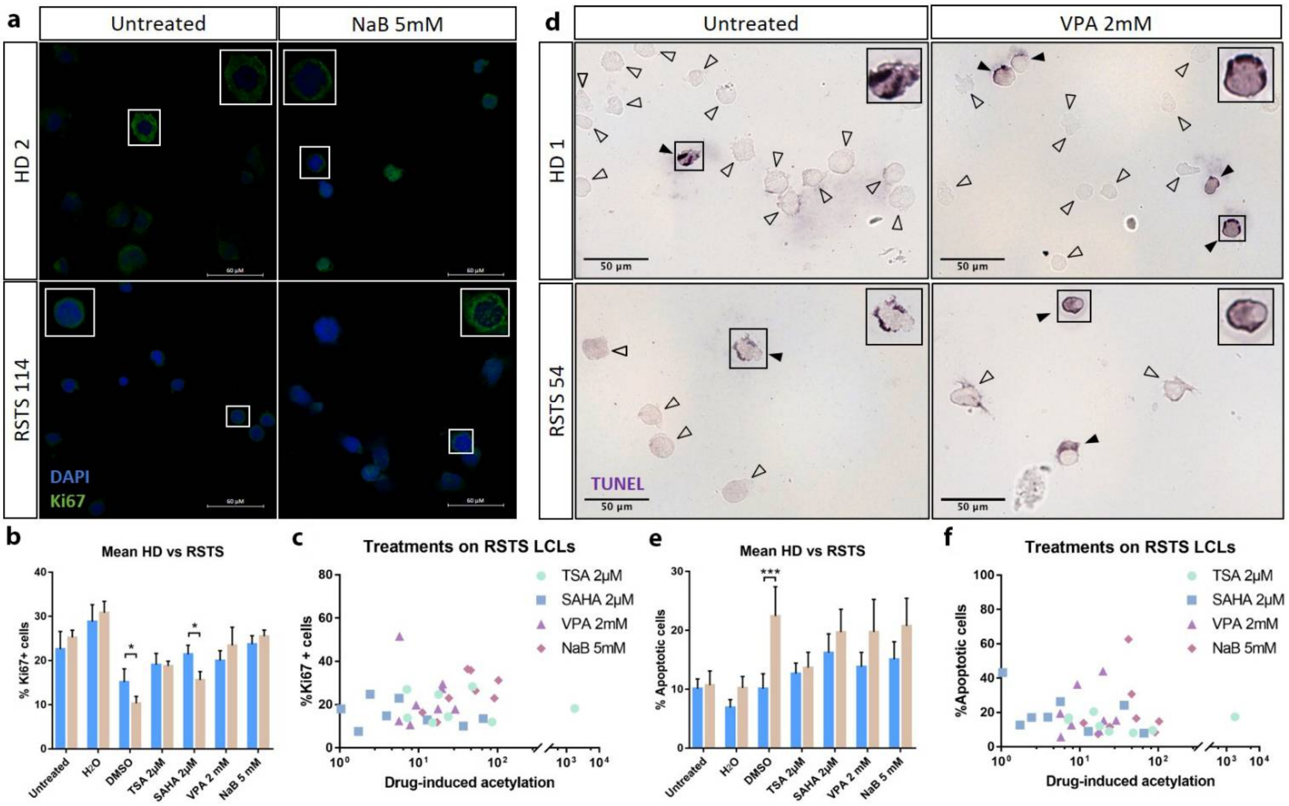

Figure S2. HDAC inhibitors cytotoxicity analysis on RSTS LCLs. Cell proliferation **(a-c)** and cell death **(d-f)** in RSTS LCLs compared to HD LCLs. **a)** Confocal 60x images showing an example of Ki67 assay performed on HD LCL (HD2) and RSTS LCL (RSTS 114) untreated and after exposure to NaB 5mM; nuclei are marked with DAPI (blue) and proliferative cells with Ki67 (green signal); Insets show 100x cell magnification. **b)** Cell proliferation rate of Ki67 positive cells (% Ki67+ cells, on Y-axis, ±SD) of RSTS LCLs compared to HD LCLs upon HDACi exposure (TSA 2μM, SAHA 2μM, VPA 2mM and NaB 5mM), treatment with vehicles (H_2O and DMSO) and untreated condition (X-axis); no significant differences in proliferation rate were detected except for exposure to vehicle DMSO and SAHA 2μM ($p<0.05$) compared to HD LCLs. **c)** Correlation overview between cell proliferation rate (% Ki67+ cells, on Y-axis) and drug-induced acetylation (X-axis) in RSTS LCLs exposed to different HDACi (TSA 2μM, SAHA 2μM, VPA 2mM and NaB 5mM), with no significant correlation disclosed between the two parameters (Pearson correlation $p>0.05$). **d)** Brightfield 40x acquisitions showing an example of TUNEL assay performed on HD LCL (HD1) and RSTS LCL (RSTS 54) untreated and after exposure to VPA 2mM, with apoptotic cells appearing deep purple (TUNEL positive and negative cells are pointed respectively with black and empty arrowheads); Insets show 80x cell magnification. **e)** Cell death rate of TUNEL positive cells (% Apoptotic cells, on Y-axis, ±SD) of RSTS LCLs compared to HD LCLs upon HDACi exposure (TSA 2μM, SAHA 2μM, VPA 2mM and NaB 5mM), treatment with vehicles (H_2O and DMSO) and untreated condition (X-axis); as expected, significant differences in cell death for patients LCLs exposed to DMSO were observed compared to HD LCLs ($p<0.001$). **f)** Correlation overview between cell death rate (% Apoptotic cells, on Y-axis) and drug-induced acetylation (X-axis) in RSTS LCLs exposed to different HDACi (TSA 2μM, SAHA 2μM, VPA 2mM and NaB 5mM), showing no significant Pearson correlation p value. Cell proliferation and cell death rate groups were compared using Student's *t-test* as statistical method (*$p<0.05$; **$p<0.01$; ***$p<0.001$).

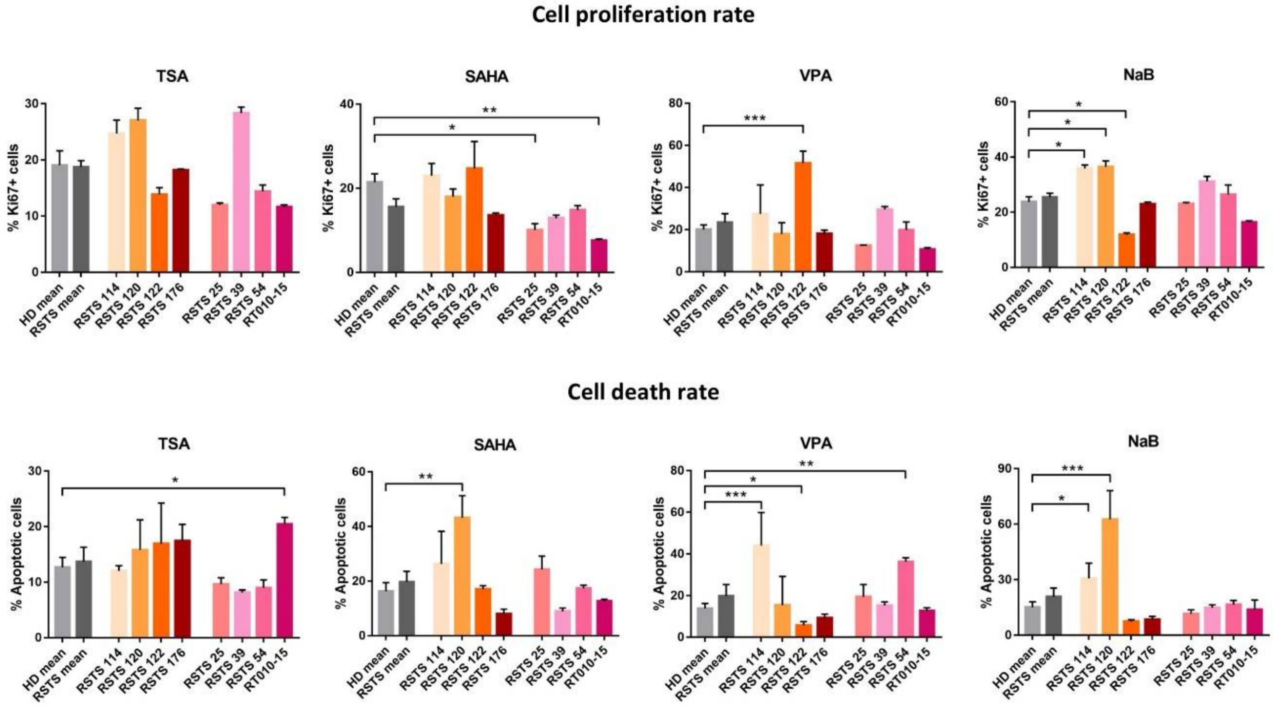

Figure S3. Insights on cell proliferation and cell death rate of RSTS LCLs upon HDAC inhibitors exposure.

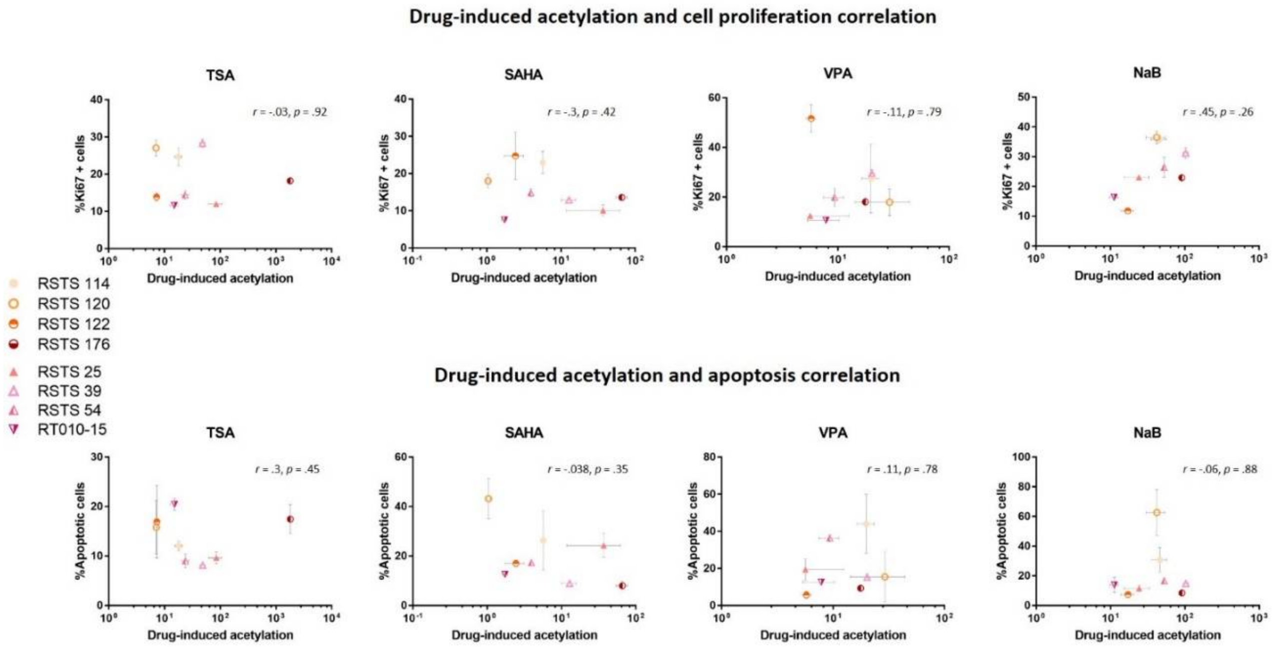

Figure S4. Correlation between HDACi-induced acetylation versus cell proliferation and apoptosis in RSTS LCLs. Correlation between cell proliferation rate (% Ki67+ cells, on Y-axis) and drug-induced acetylation (X-axis) in RSTS LCLs exposed to different HDACi (TSA 2μM, SAHA 2μM, VPA 2mM and NaB 5mM) was not significant (Pearson correlation $p>0.05$): treatments with TSA 2μM and VPA 2mM showed a very weak negative correlation ($r=-0.03$ and $r=-0.11$ respectively), SAHA 2μM a weak negative correlation ($r=-0.3$), while NaB 5mM a moderate positive correlation ($r=0.45$). Correlation between cell death rate (% Apoptotic cells, on Y-axis) and drug-induced acetylation (X-axis) in RSTS LCLs exposed to different HDACi (TSA 2μM, SAHA 2μM, VPA 2mM and NaB 5mM) showed no significant Pearson correlation p value: TSA 2μM and VPA 2mM showed, respectively, a weak and a very weak positive correlation ($r=0.3$ and $r=0.11$), while SAHA 2μM and NaB 5mM shared a very weak negative correlation ($r=-0.038$ and $r=-0.06$ respectively).

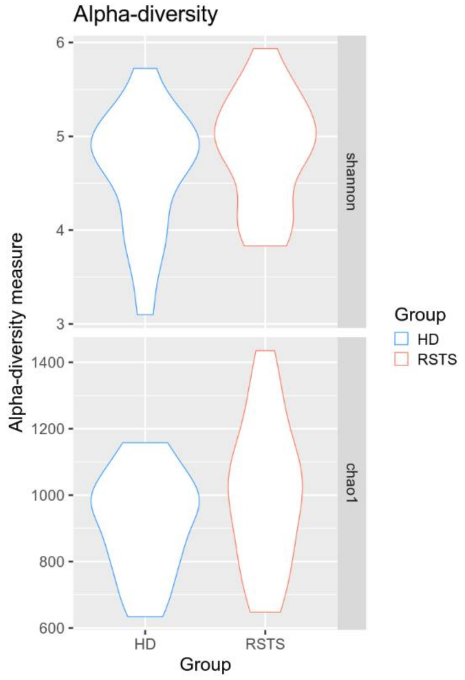

Figure S5. Gut microbiota composition in HD and RSTS subjects.

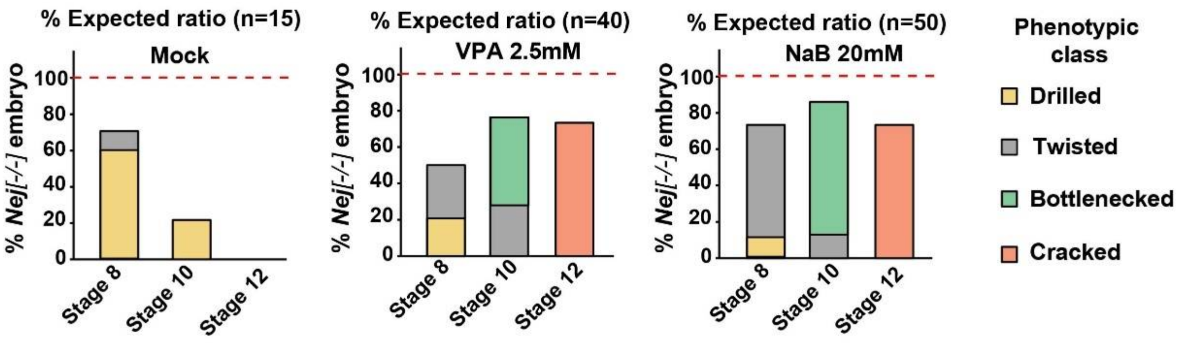

Figure S6. Normal and altered phenotypes of *nej* mutant embryos from stage 8 to 12 treated or not with HDACi.

Table S1. RSTS LCLs used for *in vitro* treatments.

Gene	RSTS LCLs	cDNA change	Protein change	Mutation type	Reference
CREBBP	RSTS 114	c.4485-7G>C	p.(R1428_G1465del)<br>p.(F1379_G1465del)	Splicing	Lopez-Atalaya et al., 2012
	RSTS 120	c.5837dupC	p.(P1947Tfs*19)	Frameshift	Spena et al., 2015
	RSTS 122	c.4394+5G>T	p.(R1428_G1465del)<br>p.(F1379_G1465del)	Splicing	Spena et al., 2015
	RSTS 176	c.4508A>T	p.(Y1503F)	Missense (HAT)	Spena et al., 2015
EP300	RSTS 25	c.41_51delinsT	p.(K14Ifs*31)	Frameshift	Negri et al., 2015
	RSTS 39	c.4640dupA	p.(N1547Kfs*3)	Frameshift	Negri et al., 2016
	RSTS 54	c.669dupT	p.(Q223Sfs*19)	Frameshift	Negri et al., 2015
	RT010-15	c.4763T>C	p.(M1588T)	Missense (HAT)	this study

Table S2. Conditions of *in vitro* treatments used on LCLs.

Treatment	TSA	SAHA	VPA	NaB
**Against**	Class I, IIa, IIb HDAC	Class I, IIa, IIb HDAC	Class I (HDAC1, HDAC2, HDAC3)	Class I HDAC
**Vehicle**	DMSO	DMSO	H_2O	H_2O
**Time**	2h	24h	24h	24h
**Dosage**	1 - **2** - 5 μM	1 - **2** - 10 μM	0,5 - 1 - **2** mM	1 - 2 - **5** mM
**Reference**	Schölz et al., 2015; Chang et al., 2018; Freese et al., 2019	Schölz et al., 2015; Freese et al., 2019; Tarasenko et al., 2018	Schölz et al., 2015; Chang et al., 2018; Tarasenko et al., 2018; Gottlicher et al., 2001	Schölz et al., 2015; Chang et al., 2018; Chriett et al., 2019

Table S3. Nutritional values of the enrolled patients. Daily dietary intake of energy and macronutrients of in RTST patients and healthy controls; values are expressed as mean (standard deviation). p-values <0.05 are considered significant (Mann-Whitney test).

Variable	HD<br>Mean (SD)	RSTS<br>Mean (SD)	p-value	Reference values
**Energy intake**				boys:1330-4020
kcal	1528 (343)	1185 (294)	0.0054**	girls:1220-3550
				kcals (AR)
**Proteins**				
g	60.8 (17.97)	46.22 (13.21)	0.0079**	16-50 g (AR)
% energy	15.93 (3.35)	15.72 (3.29)	0.8990	12-15% (RI)
**Lipids**				
g	51.55 (15.05)	43.73 (13)	0.0609	
% energy	30.36 (6.96)	33.16 (5.38)	0.1206	20-35% (RI)
**Carbohydrates**				
g	209.4 (60.81)	158.8 (41.48)	0.0054**	
% energy	54.29 (7.62)	53.65 (5.48)	0.5626	45-60% (RI)
**Total fiber**				
g	20.41 (420.05)	17.33 (13.4)	0.4369	
g/1000 Kcal	12.87 (10.35)	14.54 (9.19)	0.2065	8.40 g/1000 kcal (AI)

AR. average requirement; RI. reference intake; AI. adequate intake.

Table S4. Gut microbiota composition in HD and RSTS subjects. Major bacterial groups were organized in three phylogenetic levels (phylum, family, genus) and reported as average relative abundance ± standard deviation. p-values <0.05 were considered significant.

TAXONOMIC LEVEL			HD	RSTS	p-value	
**Phylum**	**Family**	**Genus**				
FIRMICUTES			73.4 ± 15.6	58.5 ± 18.8	0.019	*
	Ruminococcaceae		41.9 ± 15.1	32.2 ± 13.9	0.049	*
		Faecalibacterium	9.8 ± 2.2	3.3 ± 3.8	0.001	***
		Ruminococcus	6.4 ± 5.1	6.4 ± 4.9	0.877	
		Oscillospira	2.4 ± 2.4	5.1 ± 5.0	0.007	**
		Ruminococcaceae (other)	13.3 ± 15.9	8.2 ± 10.2	0.746	
		Unclass. Ruminococcaceae	9.6 ± 9.0	9.0 ± 10.1	0.525	
	Lachnospiraceae		16.2 ± 7.2	13.1 ± 7.3	0.187	
		Roseburia	5.2 ± 5.8	3.4 ± 4.9	0.053	
		Blautia	2.5 ± 3.3	1.8 ± 1.3	0.855	
		Coprococcus	2.2 ± 1.4	2.0 ± 2.4	0.168	
		Clostridium	1.1 ± 1.6	0.6 ± 1.1	0.263	
		Dorea	0.8 ± 0.9	0.8 ± 1.0	0.855	
		Unclass. Lachnospiraceae	3.3 ± 3.4	2.7 ± 2.1	0.471	
	Veillonellaceae		6.0 ± 6.1	5.1 ± 5.4	0.703	
		Dialister	5.1 ± 5.9	3.1 ± 4.9	0.501	
	Clostridiaceae		2.4 ± 3.8	0.9 ± 1.2	0.095	
		Clostridium	1.1 ± 1.6	0.6 ± 1.1	0.263	
	Unclassified Clostridiales		4.8 ± 6.7	3.6 ± 5.9	0.746	
	Streptococcaceae		1.0 ± 2.0	1.8 ± 2.7	0.315	
		Streptococcus	1.0 ± 2.0	1.7 ± 2.7	0.641	

BACTEROIDETES			16.8 ± 14	28.7 ± 21	0.065	
	Bacteroidaceae		10.3 ± 10.3	21.1 ± 16.3	0.021	*
		Bacteroides	10.3 ± 10.3	21.1 ± 16.3	0.021	*
	Rikenellaceae		2.6 ± 2.5	3.7 ± 3.3	0.220	
		Unclass. Rikenellaceae	2.5 ± 2.4	3.6 ± 3.3	0.263	
	Prevotellaceae		2.0 ± 4.4	0.9 ± 3.0	0.110	
		Prevotella	2.1 ± 4.4	0.8 ± 3.0	0.115	
	Porphyromonadaceae		0.8 ± 1.4	1.6 ± 2.2	0.177	
		Parabacteroides	1.4 ± 2.2	1.5 ± 2.3	0.217	
VERRUCOMICROBIA			6.8 ± 14.7	9.4 ± 10.1	0.056	
	Verrucomicrobiaceae		6.8 ± 14.7	9.4 ± 10.1	0.056	
		Akkermansia	6.8 ± 14.7	9.4 ± 10.1	0.056	
PROTEOBACTERIA			1.2 ± 1.5	2.1 ± 2.1	0.061	
	Enterobacteriaceae		1.0 ± 1.5	1.5 ± 2.2	0358	
		Escherichia	0.8 ± 1.2	1.3 ± 2.2	0.263	
ACTINOBACTERIA			1.6 ± 2.2	1.1 ± 1.9	0.621	
	Bifidobacteriaceae		1.4 ± 2.2	1.0 ± 1.9	0.724	
		Bifidobacterium	1.4 ± 2.2	1.1 ± 1.9	0.724	
